# Functional Characterization of Two Thioredoxin Proteins of *Toxoplasma gondii* Using the CRISPR-Cas9 System

**DOI:** 10.3389/fvets.2020.614759

**Published:** 2021-01-14

**Authors:** Zhi-Wei Zhang, Ting-Ting Li, Jin-Lei Wang, Qin-Li Liang, Hai-Sheng Zhang, Li-Xiu Sun, Xing-Quan Zhu

**Affiliations:** ^1^State Key Laboratory of Veterinary Etiological Biology, Key Laboratory of Veterinary Parasitology of Gansu Province, Lanzhou Veterinary Research Institute, Chinese Academy of Agricultural Sciences, Lanzhou, China; ^2^College of Veterinary Medicine, Shanxi Agricultural University, Taigu, China; ^3^Key Laboratory of Veterinary Public Health of Higher Education of Yunnan Province, College of Veterinary Medicine, Yunnan Agricultural University, Kunming, China

**Keywords:** *Toxoplasma gondii*, CRISPR-Cas9, thioredoxin, functional characterization, toxoplasmosis

## Abstract

Toxoplasmosis caused by infection with *Toxoplasma gondii* is an important parasitic zoonosis with a worldwide distribution. In this study, we examined the functions of two thioredoxins (namely CTrp26 and CTrx1) of *T. gondii* tachyzoites by generation of HA tag strains or gene deficient parasites in Type I RH strain (ToxoDB#10). Immunofluorescence analysis (IFA) was used to investigate the subcellular localization of the thioredoxins (Trxs). Results of IFA showed that both CTrp26 and CTrx1 were located in the cytoplasm of *T. gondii*. Functional characterizations of CTrp26 and CTrx1-deficient parasites were performed by plaque assay, intracellular replication, egress, H_2_O_2_ resistance, detection of reactive oxygen species (ROS) level and total antioxidant capacity (T-AOC) assays *in vitro*, as well as mouse infection *in vivo*. Our results showed that deletion of CTrp26 or CTrx1 did not influence the ability of *T. gondii* RH strain to replicate, egress, form plaque, resist H_2_O_2_ exposure, maintain the ROS level, and T-AOC, and also did not serve as virulence factors in Kunming mice. Taken together, these results provide new properties of the two Trxs. Although they are not essential for RH strain, they may have roles in other strains of this parasite due to their different expression patterns, which warrants future research.

## Introduction

*Toxoplasma gondii* is an obligate parasite that belongs to the apicomplexa with a worldwide distribution (1). This parasite can infect almost all warm-blooded animals and ~1/3rd of the world population are seropositive (2). Horizontal transmission of *T. gondii* occurs via consumption of raw or undercooked meat containing tissue cysts or by ingestion of water, food, and soil contaminated with oocysts (1, 3). In immunologically healthy subjects, most people are asymptomatic (2). However, in immunocompromised individuals, especially in patients with AIDS and organ transplants, the symptoms that toxoplasmosis causes are severe, or even life-threatening (4). Toxoplasmic encephalitis is the primary manifestation in these patients, accompanied by headache, ataxia, loss of memory, fever, and other symptoms (1, 5). In addition, *T. gondii* can be transmitted via vertical transmission (6). It is noticeable that the placenta is not only a barrier to protect fetus but also a target organ where *T. gondii* multiplication occurs. Once transplacental transmission occurs in the first and second trimesters, it leads to severe influence on the growth of fetus, probably resulting in microcephalus, hydrocephalus, cataract, strabismus, retinochoroiditis even abortion (1, 2, 7).

As an aerobiont, *T. gondii* can multiply rapidly within host cells at the infective stage of tachyzoites. To survive, *T. gondii* must undergo the redox stress which is induced by the microenvironment of host cells. This parasite not only eliminates oxidized material activity, but also resists the damage of reactive oxygen species (ROS) generated by host cells (8, 9). Thus, there should be a variety of mechanisms to balance the redox state in *T. gondii*, which is hypersensitive to redox imbalance.

Several peroxidases of *T. gondii*, including catalase, peroxiredoxin, superoxide dismutase (SOD), glutathione peroxidase, and thioredoxin (Trx) peroxidase, constitute the antioxidant network to protect this parasite from damage of ROS (9). *T. gondii* uses catalase to eliminate the by-products of peroxisome, such as H_2_O_2_. Peroxiredoxin can assist catalase to detoxify ROS, and it also protects *T. gondii* against H_2_O_2_ stress (10). There are two classical antioxidant systems in this parasite, namely glutaredoxin (Grx) system and thioredoxin (Trx) system, which serve as thiol-disulfide pairs to control the redox balance of *T. gondii* (9, 11). Glutaredoxin, glutathione (GSH), nictinamide adenine dinucleotide phosphate (NADPH), and glutathione reductase constitute one of the classical antioxidant systems, namely Grx system (9, 12). After reacting with target protein that contains disulfide bond, Grx becomes oxidized form, and then is reduced by GSH. NADPH and glutathione reductase operate together to reduce GSSG that means oxidized GSH, to become reduced form (13). Some Grx, such as human Grx2, can utilize electrons supplying from TrxR to keep redox balance (14). The basic biological functions of Grx are to maintain the ratio of GSH/GSSG at the normal level and the high concentration of thiol groups within cells, which overlaps the function of Trx to some extent (15). Thioredoxin system consists of thioredoxin (Trx), thioredoxin reductase (TrxR) and NADPH, which is ubiquitous in eukaryotes and prokaryotes (16). Oxidized thioredoxin, Trx-S_2_, forms after thiol-disulfide exchange reactions occurred *via* Trx-(SH)_2_ and oxidized proteins, and Trx-S_2_ is reduced by TrxR and NADPH that serves sufficient hydrogen electrons. Three components of this system participate mutually in several biological activities to protect organisms from oxidized damage and apoptosis (16, 17). The physiological function of Trx in virtually all organisms is to keep cytoplasmic proteins with reduced form, which means relatively stable (18).

The physiological functions of Trxs may be multiple in different organisms, evolving from catalyzing thiol-disulfide exchange reaction to specialized functions. It seems that Trx is “moonlighting protein,” and the functions of Trxs in *T. gondii* are still unclear. Here, we used the CRISPR-Cas9 technique to study two Trx genes, namely CTrp26 (TGME49_290260) and CTrx1 (TGME49_266620), in the virulent *T. gondii* RH strain. The subcellular localization of the two Trxs, the abilities of Trx-deficient tachyzoites to form plaque, replicate, egress, resist H_2_O_2_ and maintain ROS level and total antioxidant capacity (T-AOC) *in vitro* and the virulence in mice *in vivo* were evaluated.

## Materials and Methods

### Parasite and Cell Cultures

The tachyzoites of *T. gondii* RH strain (Type I, ToxoDB#10) were maintained *in vitro* in human foreskin fibroblast (HFF, ATCC®, SCRC-1041^TM^) at 37°C,5% CO_2_ atmosphere. HFF cells were cultured in Dulbecco's Modified Eagle medium (DMEM) supplemented with 2% fetal bovine serum (FBS), 10 mM HEPES (pH 7.2), 100 Ug/ml streptomycin, and 100 U/ml penicillin (19). Infected cells were cracked by 27-gauga needle to release tachyzoites and then filtered using Millipore filter of 5 μm pore size.

### Construction of Trx Mutant Strains

Trx knockout strains were generated using CRISPR-Cas9 approach as previously described (19). All primers used in this study are listed in Supplementary Table 1. Briefly, SgRNA of each Trx was inserted into the plasmid pSAG1-Cas9-SgUPRT to replace the UPRT targeting RNA using a Q5 Mutagenesis Kit (NEB). After sequencing, positive plasmids were extracted by using an Endo-Free Plasmid DNA Mini Kit (Omega). The 5′ and 3′ homologous arms of each Trx were amplified from the genomic DNA of *T. gondii* RH strain with each pair of specific primers listed in Supplementary Table 1. The plasmid pUPRT-DHFR-D was used as the template to generate DHFR fragment. Then three fragments mentioned above were engineered into the plasmid pUC19 with multi-fragment cloning method using a CloneExpress II one-step Cloning Kit (Vazyme). Positive plasmids were used to amplify the fragment of 5HR-DHFR-3HR to generate the homologous templates. About 40 μg positive plasmids of CRISPR-Cas9 and 15 μg purified fragment of 5HR-DHFR-3HR templates were co-transfected into tachyzoites of *T. gondii* RH strain by electroporation. Single strain clones were obtained by limiting dilution method and cultured in 96-well-plates with 3 μM pyrimethamine. Trx knockout strains were confirmed by PCR and RT-PCR as previously described (20).

### C-Terminal Tagging

The CRISPR-Cas9 plasmids of each Trx that targets the 3′ region of each gene were constructed as previously described (21). The PCR product of each Trx that contains ~1.5 kb of 3′ region of a Trx gene (except the STOP codon) and the fragment of 10×HA and DHFR was amplified from the p10×HA-LIC-DHFR plasmids as the template using a pair of specific primers. The purified fragment and C-terminal Trx-specific CRISPR-Cas9 plasmids were mixed and co-transfected into freshly egressed tachyzoites of *T. gondii* RH strain using electroporation. Single positive clones were identified by PCR and immunofluorescence analysis (IFA).

### Western Blotting and Immunofluorescence Analyses

For Western blotting, freshly purified tachyzoites of *T. gondii* RH strain were lysed by RIPA lysis buffer on ice to obtain proteins and were used for SDS-PAGE analysis, then transferred to polyvinylidene fluoride (PVDF) member. The primary antibodies used in Western blotting were rabbit anti-Aldolase (1:500) and rabbit anti-HA (1:1,000) (21).

For immunofluorescence analysis (IFA), HFF cells were infected with freshly egressed tachyzoites of *T. gondii* RH strain and were fixed with 4% paraformaldehyde, then penetrated with 0.2% Triton X-100 for 20 min. After blocked by 5% BSA diluted in PBS for 1 h, infected cells were incubated with rabbit anti-IMC1 (1:1,000) and mouse anti-HA (1:500) as primary antibodies at 4°C for overnight, and then incubated with Alexa Fluor 488 anti-rabbit IgG (1:1,000) and Alexa Fluor 594 goat anti-mouse IgG (1:1,000) as secondary antibodies at 37°C for 1 h. The samples were imaged using a Leica confocal microscope system (tcs sp52, Leica, Germany) (21).

### Plaque Assay

About 200 tachyzoites of Trx knockout and wild-type (WT) RH strains were inoculated into 12-well-plates containing monolayer of HFF cells, and the 12-well-plates were inoculated for 1 week under 37°C, 5% CO_2_ environment. The medium was removed and the infected cells were fixed with 4% paraformaldehyde for 30 min and stained with 0.5% crystal violet for 10 min at ambient temperature. The number and size of plaques were counted and analyzed (22).

### Intracellular Replication

Tachyzoites of *T. gondii* RH strain were incubated into cell culture dishes containing monolayer of HFF cells for 1 h, with about 10^5^ tachyzoites of each Trx knockout or WT strains per dish. After removing old medium, the dishes were washed three times to remove extracellular parasites. Then, infected cells were added with new culture medium and maintained at 37°C with 5% CO_2_ atmosphere for 23 h. HFF cells were fixed with 4% paraformaldehyde for 30 min and stained with mouse anti-SAG1, followed by Alexa Fluor 594 goat-anti mouse IgG. Parasitophorous vacuoles (PVs) were selected randomly for analysis, and the numbers of parasites contained in PVs were recorded (22).

### Egress Assay

Cell culture dishes containing monolayer of HFF cells were inoculated with freshly harvested tachyzoites of Trx knockout or WT strains, with about 10^5^ tachyzoites per dish, and were maintained at 37°C with 5% CO_2_ for 1 h. Then the dishes were washed three times with PBS to remove floating parasites and added with fresh culture medium to continue to culture for further 30–36 h. Then infected cells were treated with 3 μM calcium ionophore A23187 diluted in DMEM. The timing and images of parasites to egress from host cells were recorded by live cell microscopy (20).

### H_2_O_2_ Resistance Analysis

Freshly egressed tachyzoites were cultured in regular DMEM or DMEM containing 1.6 mM H_2_O_2_ for 1 h at 37°C, 5% CO_2_ atmosphere, then centrifuged at 1,000 g to remove the supernatants. Parasites were re-suspended in DMEM and inoculated into 12-well-plates containing monolayer of HFF cells to culture for 40 h. After being fixed with 4% paraformaldehyde, the samples were stained with mouse anti-SAG1, followed by Alexa Fluor 594 goat-anti mouse IgG. PVs were selected to count the number of parasites that PVs contained.

### Detection of ROS Level in Trx Mutant Strains

Infected cells were cracked by 27-gauga needle to release tachyzoites. 1 × 10^6^ purified tachyzoites were labeled with DCFH-DA (Beyotime, China) and suspended in DMEM. After incubated darkly at 37°C, 5% CO_2_ atmosphere for 1 h, samples were centrifuged at 1,200 g to remove the supernatants and washed three times with PBS. Total fluorescence intensity of tachyzoites were determined with fluorescence microplate reader (Spectra Max M5, MD, USA) with 488 nm of excitation wavelength and 525 nm of emission wavelength.

### Analysis of Total Antioxidant Capacity

Purified tachyzoites were resuspended in PBS and broken with ultrasonic cell disruptor (Uibra-Cell, Sonics, USA). The suspensions were centrifuged at 12,000 g under 4°C to extract proteins of *T. gondii*. T-AOC was measured by T-AOC assay kit (Beyotime, China) according to the manufacturer's instructions. The results were represented as total antioxidant capacity in per gram of protein.

### Mouse Infection With Trx- Deficient Strains

Specific-pathogen-free (SPF) Kunming mice (female, 7 weeks old) were purchased from The Center of Laboratory Animals, Lanzhou Veterinary Research Institute, Chinese Academy of Agricultural Sciences. Mice (5 mice/cage) were habituated for 1 week to reduce stress response before experiment. Mice were infected with freshly egressed tachyzoites of Trx knockout or WT strains (10 mice/strain) by intraperitoneal injection, with 100 tachyzoites of *T. gondii* RH strain per mouse. All mice were recorded daily for the progression of illness and death time.

### Statistical Analysis

The differences of experiments *in vitro* and *in vivo* were analyzed using Student's *t*-test and Gehan–Breslow–Wilcoxon test with GraphPad Prism 5 (GraphPad Software, La Jolla, CA, USA). Data obtained from three independent experiments were presented as means ± standard deviations (SD). The differences were considered statistically different when *P*-value was below 0.05.

## Results

### Localization of CTrp26 and CTrx1

**Figure 1:**
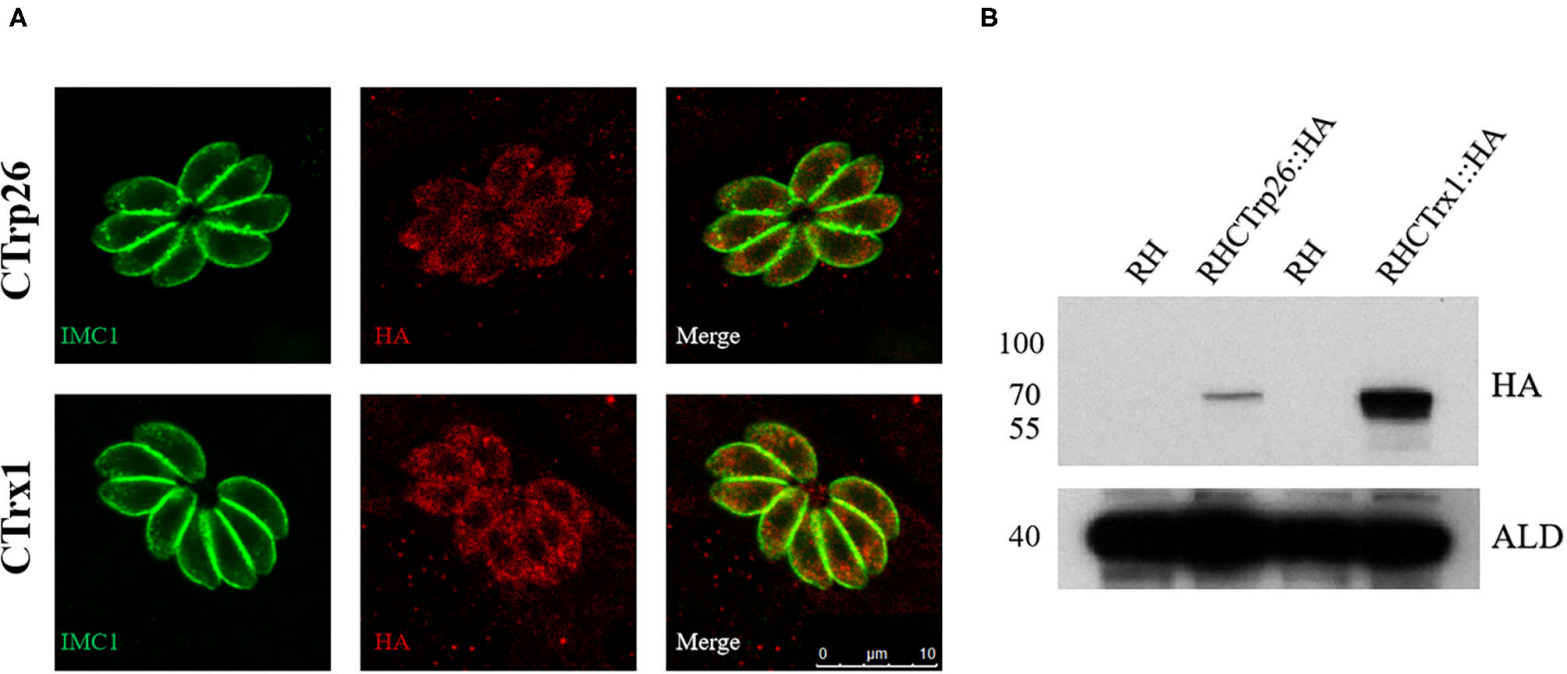
Immunofluorescence analysis and Western blotting of CTrp26 and CTrx1. **(A)** Immunofluorescence analysis shows that both CTrp26 and CTrx1 are located in cytoplasm of *Toxoplasma gondii*. **(B)** Western blotting confirmed that the 10×HA were successfully inserted into the C terminal of CTrp26 or CTrx1. The 10×HA tagged CTrp26 and CTrx1 are about 62.3 and 61.7 kDa, respectively. Anti-adolase (ALD) was served as a loading control.

To determine the subcellular localization of CTrp26 and CTrx1 in *T. gondii*, 10×HA (~39 kDa) was inserted into the C-terminus of CTrp26 or CTrx1 gene endogenous locus. IFA results showed that CTrp26 and CTrx1 were both located in the cytoplasm of *T. gondii* (Figure 1A). The findings were consistent with the results of Western blotting analysis, with the bond of CTrp26 and CTrx1 occurred between 55 and 70 kDa (Figure 1B).

### Construction of RHΔCTrp26 and RHΔCTrx1 Strains by CRISPR-Cas9

**Figure 2:**
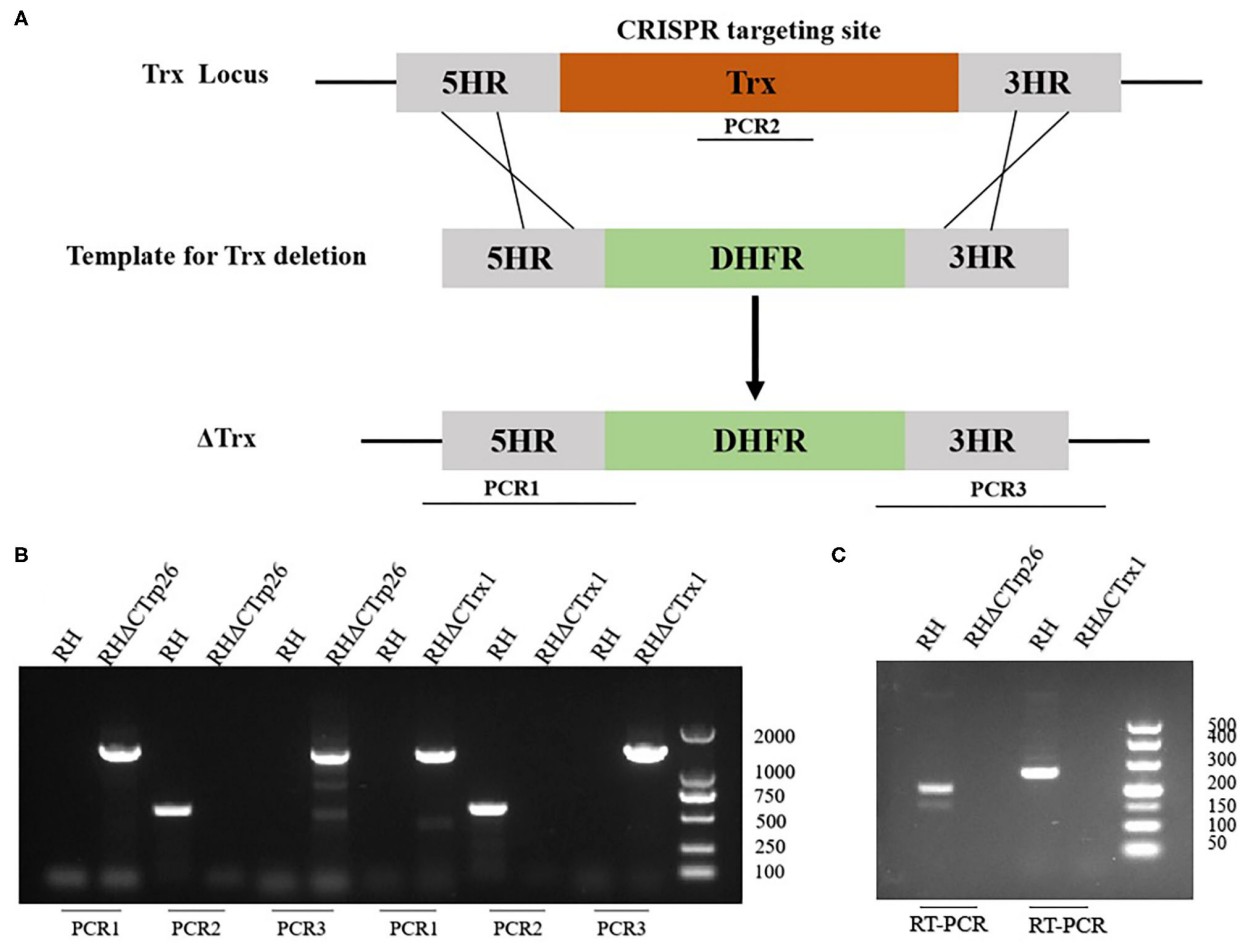
Construction of CTrp26 or CTrx1 knockout strains. **(A)** Schematic representation of deleting CTrp26 or CTrx1 by CRISPR-Cas9-mediated homologous replacement. **(B)** Diagnostic PCRs show that CTrp26 or CTrx1 gene was successfully disrupted, which is confirmed by at mRNA level **(C)**.

CRISPR-Cas9 technique was used to delete the CTrp26 and CTrx1 genes in type I RH strain, and CTrp26 or CTrx1 coding region was replaced by 5HR-DHFR-3HR fragment with homologous recombination technology (Figure 2A). Single clones obtained from drug selection and limiting dilution methods were confirmed by PCR, and ~600 bp fragment was amplified in the WT strain but was not detected in KO strains because of the replacement by the 5HR-DHFR-3HR fragment (Figure 2B). RT-PCR method was also used to confirm the deletion of CTrp26 or CTrx1 gene at the mRNA level, with ~200 bp fragment detected in the WT strains and no fragment found in KO strains (Figure 2C). Our results showed that CTrp26 and CTrx1 genes were disrupted by CRISPR-Cas9-mediated homologous recombination technology and that RHΔCTrp26 and RHΔCTrx1 strains were successfully constructed.

### Disruption of CTrp26 or CTrx1 Gene Did Not Affect the Growth and Egress of *T. gondii*

**Figure 3:**
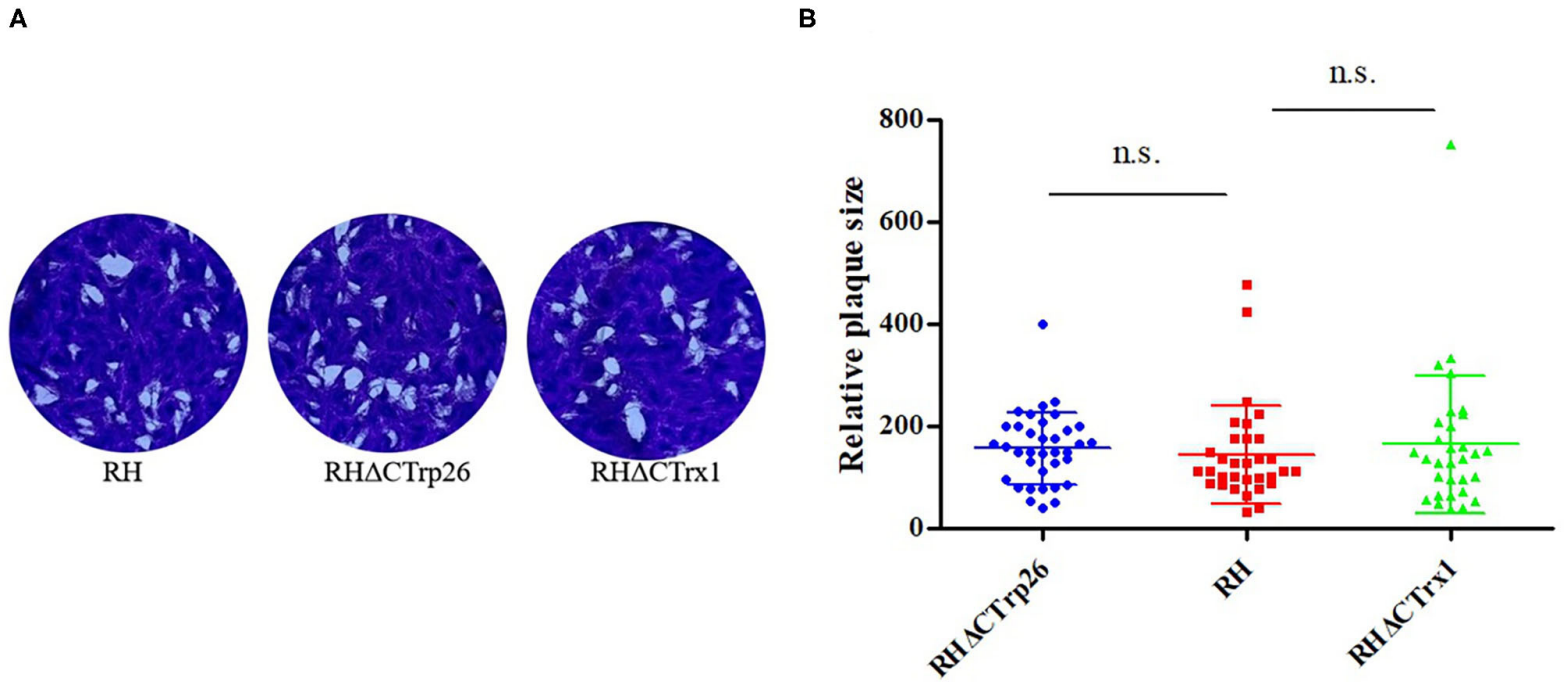
The lytic cycle of RHΔCTrp26 or RHΔCTrx1 knockout strains and WT strain *in vitro*. **(A)** Plaque assay of Trx mutant strains and WT strain. **(B)** Relative size of the plaques generated by Trx mutant strains and WT strain showed no significant differences between WT strain and any of Trx mutant strains. n. s, not significant.

Plaque assay was used to compare the ability of plaque formation between RHΔCTrp26 or RHΔCTrx1 knockout strains and WT strains (Figure 3). HFF cells grown in 12-well-plate were infected with about 200 tachyzoites of Trx mutants or WT strains to allow parasites to grow for 7 days, and the size and number of plaques were analyzed. Results showed that there were no significant differences in the size and number of plaques between cells infected with RHΔCTrp26 or RHΔCTrx1 knockout tachyzoites and WT strains.

**Figure 4:**
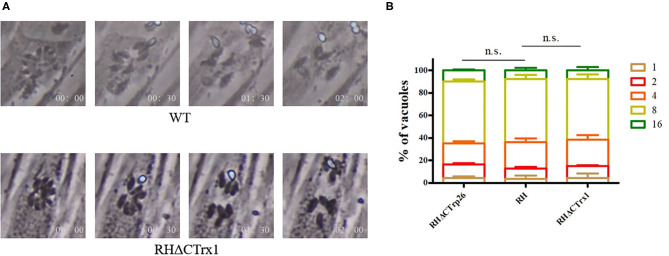
Egress process and intracellular replication of CTrp26 or CTrx1 knockout strains and WT strain *in vitro*. **(A)** Representative image shows that one of Trx mutant strains (Δ*CTrx1*) and WT strain egress within 2 min after addition of 3 μM calcium ionophore A23187. **(B)** WT strain and Trx mutant strains have similar intracellular replication dynamic. n. s, not significant.

Subsequently, we investigated the role of Trxs on egress process of *T. gondii*, 3 μM calcium ionophore A23187 were used to treat HFF cells infected with RHΔCTrp26 or RHΔCTrx1 knockout tachyzoites and WT strains, and the egress process was recorded by time-lapse microscopy over 5 min (Figure 4A). The results showed that most Trx mutants and WT strains egressed from host cells within 2 min, with no significant differences in egress process being observed among different strains.

To evaluate the effect of Trxs on the intracellular replication of *T. gondii*, Trx mutants, and their parental strains were incubated into cell dishes containing monolayer of HFF cells for 24 h, and were fixed with 4% paraformaldehyde to count tachyzoite numbers that PVs contained (Figure 4B). Results showed that the ability of intracellular growth of RHΔCTrp26 or RHΔCTrx1 knockout strains and their parental strains was similar.

### Deletion of CTrp26 or CTrx1 Gene Did Not Affect the Sensitivity of *T. gondii* to H_2_O_2_ Exposure

**Figure 5:**
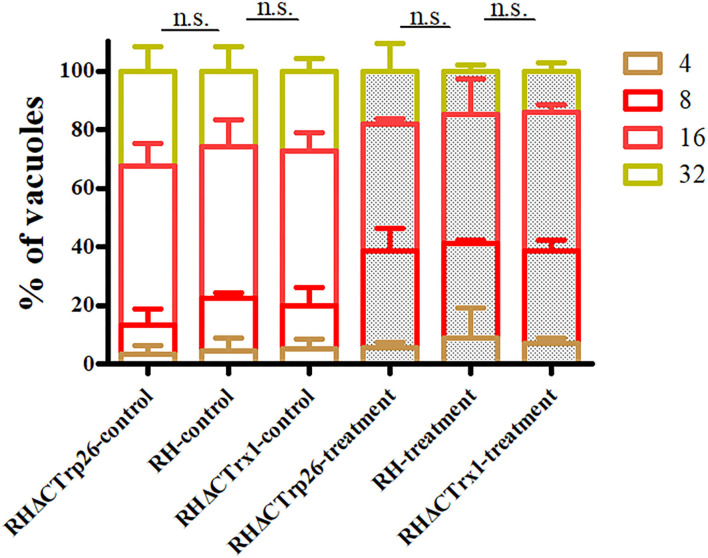
Effect of H_2_O_2_ on *T. gondii* proliferation associated with RHΔCTrp26 or RHΔCTrx1 knockout strains and WT strain *in vitro*. Control and treatment represent parasites treated with regular DMEM and DMEM containing H_2_O_2_, respectively. n. s, not significant.

Extracellular parasites treated with regular DMEM or DMEM containing H_2_O_2_ were inoculated into HFF monolayers to examine whether RHΔCTrp26 and RHΔCTrx1 knockout strains were more sensitive to oxidative stress compared with WT RH strain. The results showed that the proliferation rates of the three strains were similar under the same condition (control group or treatment group) (Figure 5); however, the ability of intracellular growth associated with treatment group was significantly lower than that in control group (*P* < 0.05), suggesting that oxidative stress inhibited the proliferation of tachyzoites, but RHΔCTrp26 and RHΔCTrx1 strains were not more sensitive to H_2_O_2_ exposure compared with WT RH strain.

### Analysis of ROS Level and T-AOC in RHΔCTrp26 and RHΔCTrx1 Strains

**Figure 6:**
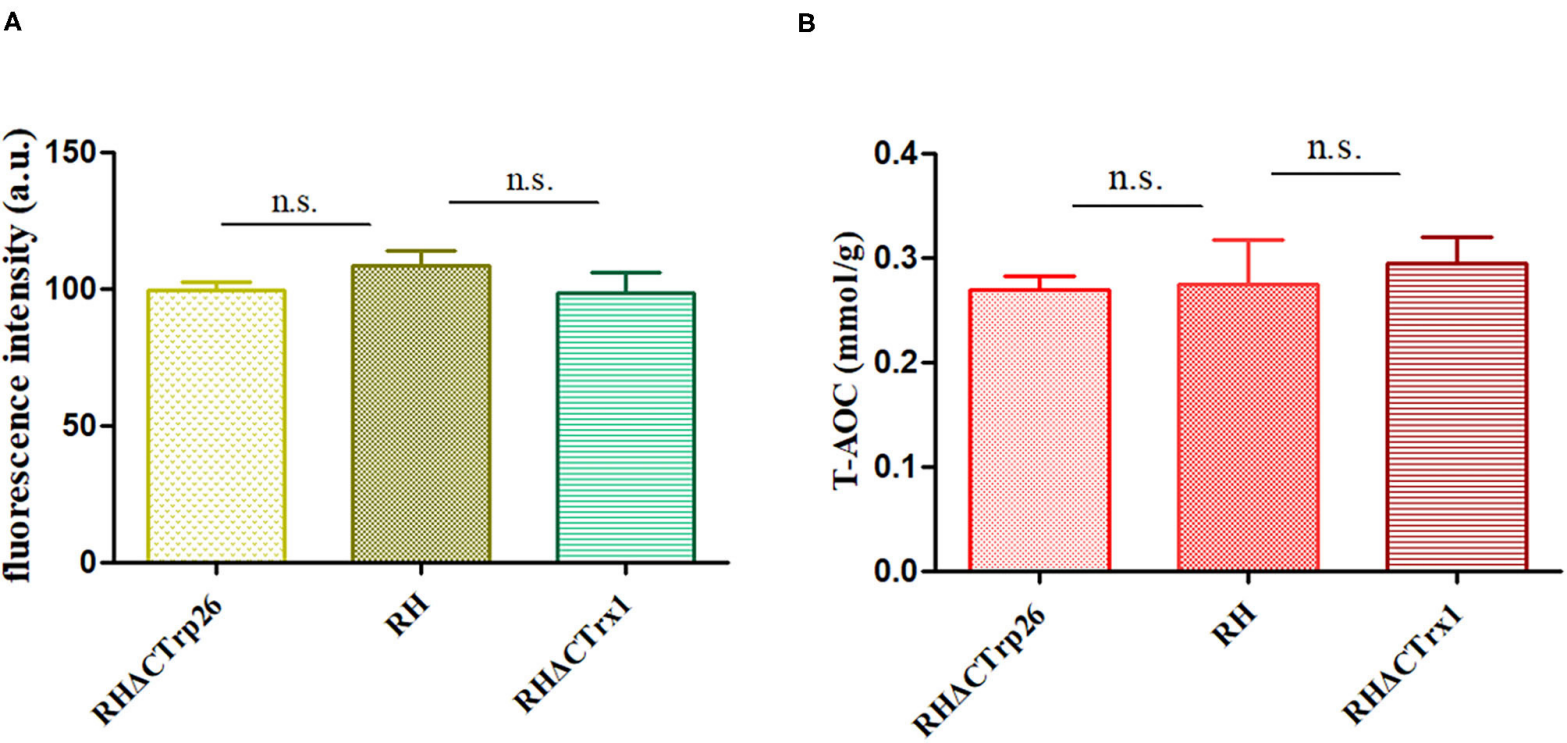
Responses of oxidative stress on RHΔCTrp26 or RHΔCTrx1 knockout strains *in vitro*. **(A)** The reactive oxygen species level in RHΔCTrp26 or RHΔCTrx1 knockout strains and WT strain. **(B)** The total antioxidant capacity in RHΔCTrp26 or RHΔCTrx1 knockout strains and WT strain. n. s, not significant.

To investigate ROS level in Trx mutants and WT strains, tachyzoites of the three strains were labeled with DCFH-DA (reduced form) and measured with fluorescence intensity of DCF (oxidized form) that reflects ROS level in *T. gondii*. The results showed that there were no significant differences in ROS level among RHΔCTrp26, RHΔCTrx1, and WT strains (Figure 6A), suggesting that disruption of CTrp26 or CTrx1 gene had no effect on ROS level in *T. gondii* RH strain.

Furthermore, we evaluated whether CTrp26 or CTrx1 deletion can affect the T-AOC of *T. gondii*. Extracted protein of tachyzoites was used to examine T-AOC using T-AOC assay kit (Beyotime, China). Our results revealed that T-AOC in per gram of protein among three strains were similar (Figure 6B), indicating that CTrp26 or CTrx1 plays no role in T-AOC in RH strain of *T. gondii*.

### Deletion of CTrp26 or CTrx1 Gene Did Not Attenuate the Virulence of *T. gondii* in Mice

**Figure 7:**
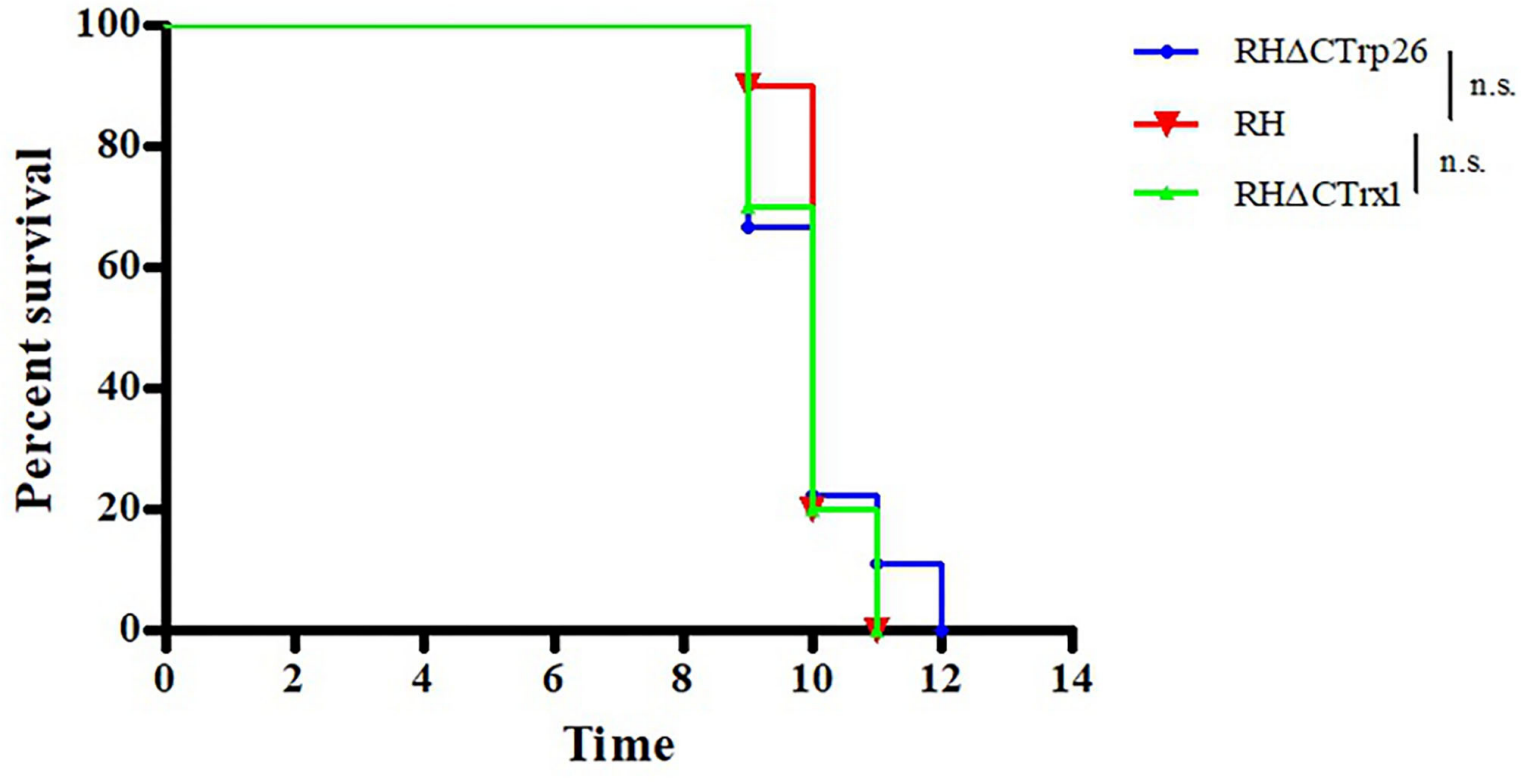
Survival of Kunming mice infected with Trx mutant strains and WT strain of *T. gondii*. The mice (10/group) were intraperitoneally injected with 100 tachyzoites of each strain. The death time of mice were recorded until all mice died.

To evaluate whether the deletion of Trxs can attenuate the pathogenicity of the parasite to mice, Kunming mice were intraperitoneally injected with 100 tachyzoites of RHΔCTrp26, RHΔCTrx1, or WT strains, and the morbidity and mortality were recorded daily (Figure 7). The results showed that all mice died within 9–12 days, suggesting that Trxs are not virulence factors and do not alter lethal action of *T. gondii* RH strain in mice.

## Discussion

Some of the Apicomplexan parasites, such as *Plasmodium falciparum* and *T. gondii*, result in severe zoonoses like malaria and toxoplasmosis (8). These parasites are aerobian protozoans that are supersensitive to the microenvironment of the host cells (8). They adapt to environmental conditions they live, thus have developed several antioxidant mechanisms to tackle the oxidative stress generated by the host cells.

There are three classic thioredoxins (PfTrx1–3) and two thioredoxin-like proteins (PfTlp1–2) that have been identified in *P. falciparum* (23). PfTrx1 is a cytoplasmic protein that can directly reduce oxidized glutathine and deloxify H_2_O_2_ to counteract oxidative stress (24). Although PfTrx2 was reported originally to locate in mitochondria, recent research revealed that PfTrx2 is the component of the translocon of exported proteins (PTEX) in PVs and involved in the protein trafficking from *P. falciparum* to host cells (25). PfTrx3 is located in the endoplasmic reticulum (ER) to fold target proteins *via* formation of disulfide band (24). However, limited studies have focused on the two thioredoxin-like proteins of *P. falciparum*, and the physiological functions of PfTlp1–2 remain unclear (23).

Catalase (CAT) is lacking in most protozoa, whereas it exists in *T. gondii* (26). A previous study indicated that CAT plays a role in the proliferation of *T. gondii* within PVs, because when CAT gene was deleted, parasites appeared more sensitive to H_2_O_2_ exposure and was less virulent to mice (27). Peroxiredoxin 2 (Prx2) is also an antioxidant enzyme that locates in the cytoplasm of *T. gondii*, it not only helps CAT to detoxify ROS, but also can eliminate H_2_O_2_ by itself (28). Prx2-overexpression parasites showed strong resistance to oxidative stress (27). In addition, there are also classical antioxidant systems in *T. gondii*, especially the thioredoxin (Trx) system, which contains three members, namely thioredoxin (Trx), thioredoxin reductase (TrxR) and nictinamide adenine dinucleotide phosphate (NADPH). The three members of the Trx system operate mutually in order to balance the redox state in *T. gondii*. However, studies of the Trx system in *T. gondii* is limited, and the functions of Trx in *T. gondii* infection remain unclear.

Only a few studies have focused on the biological functions of Trx in *T. gondii* RH strain. To date, two Trxs have been identified in *T. gondii*. TgATrx1 is located in the outermost compartment of apicoplast, cytoplasm and ER (29). TgATrx2 is an apicoplast periplastid protein, whose phenotype enrichment score is −2.87 (8, 30). Both TgATrx1 and TgATrx2 are essential for the growth of *T. gondii*, and conditional depletion of either of two Trxs leads to loss of plaque formation, which indicated that generated parasites displayed severe grow defect in the absence of TgATrx1 or TgATrx2 protein (8), suggesting that TgATrx1 is in control of protein trafficking from ER to apicoplast, and TgATrx2 affects genome copy number of apicoplast (8).

In the present study, we generated a transgenic parasite by inserting a 10×HA tag into C-terminal of CTrp26 or CTrx1 to investigate the subcellular localization of CTrp26 and CTrx1 in *T. gondii*. IFA showed that both CTrp26 and CTrx1 were located in the cytoplasm of *T. gondii* tachyzoites. Furthermore, results of Western blotting confirmed that CTrp26 and CTrx1 were expressed successfully with the HA tags.

CRISPR-Cas9 technique was used to disrupt CTrp26 and CTrx1 in type I RH strain of *T. gondii*, and RHΔCTrp26 strain and RHΔCTrx1 strain were constructed. The number and size of plaques that generated by RHΔCTrp26, RHΔCTrx1 and WT RH strains were not significantly different, suggesting that CTrp26 and CTrx1 did not play role in several lytic cycles of *T. gondii* RH strain. Furthermore, intracellular replication and egress assay revealed that the replication and egress efficiencies of CTrp26 mutant and CTrx1 mutant were comparable to that of RH WT strain. Moreover, the survival of mice was recorded in order to investigate the functions of CTrp26 and CTrx1 *in vivo*. All mice died within 9-12 days, indicating that CTrp26 and CTrx1 were not important virulence factors and deletion of either of the two genes did not attenuate the virulence of *T. gondii* RH strain in mice. The phenotype enrichment scores of CTrp26 and CTrx1 are 0.42 and 1.28, respectively, which indicates that CTrp26 and CTrx1 are not essential genes in RH strain.

Considering that the function of Trx is keeping the redox balance, we also investigated the resistance of *T. gondii* to H_2_O_2_ exposure, ROS level and T-AOC of *T. gondii*. H_2_O_2_ exposure could inhibit the proliferation of tachyzoites of RHΔCTrp26, RHΔCTrx1 mutants and RH WT strain; however, the sensitivity of the three strains was similar, suggesting that CTrp26 and CTrx1 do not play role in resistance of H_2_O_2_ damage in *T. gondii* RH strain. Our results revealed that the disruption of CTrp26 or CTrx1 in *T. gondii* RH strain did not influence the ROS level in Trx mutant strains. The deletion of CTrp26 or CTrx1 did not reduce T-AOC of *T. gondii* RH strain.

## Conclusion

Using CRISPR-Cas9 technique, the present study examined the basic biological functions of CTrp26 and CTrx1 in *T. gondii* RH strain *in vitro* and *in vivo*. Our results showed that CTrp26 and CTrx1 are both located in the cytoplasm of tachyzoites. Deletion of either of the two Trxs did not affect intracellular replication, egress process, plaque formation, H_2_O_2_ resistance, ROS level, and T-AOC of this parasite *in vitro*, and these two Trxs did not serve as virulence factors in *T. gondii* RH strain in Kunming mice. Further research will focus on the functions of CTrp26 and CTrx1 in other genotypes of *T. gondii* and the roles these two Trxs play when *T. gondii* undergo the damage of other ROS.

## Data Availability Statement

The original contributions presented in the study are included in the article/Supplementary Materials, further inquiries can be directed to the corresponding author/s.

## Ethics Statement

The animal study was reviewed and approved by the Animal Ethics Committee of Lanzhou Veterinary Research Institute, Chinese Academy of Agricultural Sciences.

## Author Contributions

J-LW and X-QZ designed the study. Z-WZ performed the experiments, analyzed the data, and wrote the manuscript. J-LW, Q-LL, and X-QZ revised the manuscript. T-TL, H-SZ, and L-XS participated in implementation of the study. All authors read and approved the final version of the manuscript.

## Conflict of Interest

The authors declare that the research was conducted in the absence of any commercial or financial relationships that could be construed as a potential conflict of interest.
