# Supplementary material for: Functional Characterization of Two Thioredoxin Proteins of Toxoplasma gondii Using the CRISPR-Cas9 System
Source: Front Vet Sci. 2021 Jan 14;7:614759. doi: 10.3389/fvets.2020.614759 (PMC7841047; doi:10.3389/fvets.2020.614759)
Supplement: Supplementary file 1 [file Table_1.DOCX]

**Supplementary Table 1 Primers used in this study**

| Primer name | Primer sequence (5′ → 3′) |
| --- | --- |
| SgCTrp26 | GAATTAGTCACCGACTTCCAGTTTTAGAGCTAGAAATAGC |
| Sg3CTrp26 | GAAGGAAATTGAAAAGGAGTGTTTTAGAGCTAGAAATAGC |
| SgCTrx1 | GATTGTCAAATGGCCCCAAGGTTTTAGAGCTAGAAATAGC |
| Sg3CTrx1 | AAGAAAATTCATTTCGTACAGTTTTAGAGCTAGAAATAGC |
| CTrp26-KOF | CTAAGCAACTCCGAGACCATT |
| CTrp26-KOR | TTTGAGGGAAGAAGAGCGTGA |
| CTrx1-KOF | ATGTGGCTTGCTGGATTTCGT |
| CTrx1-KOR | CGTAGTCAGGCATCACTCTTTGAG |
| U5CTrp26-Gbison-F | GGTTTTCCCAGTCACGACGTTTTAGTGCCTGCCTCACGGAAAA |
| U5CTrp26-Gbison-R | GGATTTACAGCCTGGCGAAGCTTGGCGACGAAACGATTGCTTGT |
| U3CTrp26-Gbison-F | CTATGCACTTGCAGGATGAATTCGAAGAAGGAAATTGAAAAGGA |
| U3CTrp26-Gbison-R | GAGCGGATAACAATTTCACAACCACGGAAACAACAAAGTAT |
| U5CTrx1-Gbison-F | GGTTTTCCCAGTCACGACGTTATTGCCTGTCCTTTGCTCACTT |
| U5CTrx1-Gbison-R | GGATTTACAGCCTGGCGAAGCTTCGTCTTCCGGTCATACACTCG |
| U3CTrx1-Gbison-F | CTATGCACTTGCAGGATGAATTCTCCCGATTCTTCTTTGACTTCTC |
| U3CTrx1-Gbison-R | GAGCGGATAACAATTTCACATCTACTTTGTATGTGGTTGCCCTA |
| CTrp26-cDNA-KOF | ATCCAGTCTTCACCTCATTGGC |
| CTrp26-cDNA-KOR | TGTTTTGAGGGAAGAAGAGCGT |
| CTrx1-cDNA-KOF | CGTCCCAAGCGGTGTCTGTTC |
| CTrx1-cDNA-KOR | ATGACGCAAAGAGTGGGCAGTG |
